# Autosomal dominant polycystic kidney disease combined with hypertrophic cardiomyopathy: A case report

**DOI:** 10.1097/MD.0000000000008625

**Published:** 2017-11-17

**Authors:** Yingjing Shen, Chenggang Xu

**Affiliations:** Department of Nephrology, Third Affiliated Hospital of Second Military Medical University, Shanghai, China.

**Keywords:** acute kidney injury, acute respiratory distress syndrome, hypertrophic cardiomyopathy, polycystic kidney disease, proteinuria

## Abstract

**Introduction:**

This report describes the novel sampling of autosomal dominant polycystic kidney disease (ADPKD) combined with hypertrophic cardiomyopathy (HCM).

**Symptoms and clinical findings:**

A 48-year-old Chinese man presented with anasarca, hypourocrinia, gross hematuria, and weight gain by 10 kg subsequently developed acute kidney injury after struck by acute respiratory distress syndrome, really a threat to his heart.

**Diagnoses:**

Abdominal ultrasound revealed multiple small cysts in both kidneys, with the right kidney measuring 11.6 cm in length, and the left kidney measuring 11.5 cm in length, which supported ADPKD. Echocardiography showed left ventricular posterior wall thickness measuring 15.2 mm, interventricular septum measuring 17.2 mm, left atrial size 31.9 mm, ejection fraction measuring 69%, approving the diagnose of HCM.

**Therapeutics interventions:**

Because of the failure treatment with tripterygium wilfordii and valsartan, the patient was administered with prednisone 1 mg/kg/day. Continuous renal replacement therapy was required to prevent heart and kidney from failure.

**Outcomes:**

The patient responded well and his renal function improved.

**Conclusion:**

This is the first reported case of ADPKD with HCM, with complete remission of acute kidney injury and preservation of cardiac function. Serial checks and measures should be considered for appropriate treatment of ADPKD patient who present with rapid decline of renal function. We present detailed analysis of the patient's disease course and review literature. Written informed consent was obtained from the patient for publication of this case report. It has been permitted by Committee on Ethics of Biomedicine, Second Military Medical University.

## 1 Introduction

Autosomal dominant polycystic kidney disease (ADPKD) is defined as an inherited disorder characterized by the growth of cysts in the kidneys and other organs. Hypertrophic cardiomyopathy (HCM) is another genetic disorder involving heart muscle.

## 2 Case report

A 48-year-old man with a history of polycystic kidney disease was admitted to Third Affiliated Hospital of Second Military Medical University on May 24, 2017. At the beginning of 2017, he sought for medical advice because of abdominal pain, and was diagnosed with intestinal obstruction. Computed tomography (CT) had scanned multiple cysts in both kidneys, and serum creatinine increased to 115 μmol/L. In April, though he had no sign of smoky urine, periorbital and extremities edema, ascites, or dyspnea, laboratory investigations showed increased serum creatinine (141 μmol/L), proteinuria (6929 mg/day), decreased serum albumin (19 g/L), and increased serum cholesterol (6.02 mmol/L). Abdominal ultrasound revealed multiple small cysts in both kidneys, with the right kidney measuring 11.6 cm in length, and the left kidney measuring 11.5 cm in length, no cyst in liver and spleen. Echocardiography showed left ventricular (LV) posterior wall thickness measuring 15.2 mm, interventricular septum measuring 17.2 mm, left atrial size 31.9 mm, ejection fraction measuring 69%.

Based on the typical CT and ultrasound findings, we diagnosed him as ADPKD, and prescribed tripterygium wilfordii (20 mg per day) and valsartan (80 mg per day). In addition, he has a history of HCM, and used to be treated by cardiac interventional therapy for recurrent arrhythmia even sudden cardiac arrest. The patient had no family history in either polycystic kidney or HCM.

Nevertheless, tripterygium wilfordii treatment proved to be failed 2 weeks later for anasarca, hypourocrinia, gross hematuria, and weight gain by 10 kg. On May 18, the patient was started on daily prednisone (60 mg/day) instead. But what's worse was that he was attacked by streptococcus pneumonia on May 29, which led to septic shock, type I respiratory failure, even acute renal injury. At that moment, his pulse oxygen was 95% (oxygen flow rate = 10 L/minute), pulse rate was 125 beats per minute and blood pressure was 90/61 mm Hg. A chest radiograph showed consolidation of right upper lung lobe. Serum biochemistry revealed a creatinine of 300 μmol/L, albumin16.4 g/L, brain natriuretic peptides 1070 pg/mL, C-reactive protein > 200 mg/L, procalcitonin 100 ng/mL, endotoxin 72.58 pg/mL, arterial partial pressure of oxygen 78.0 mm Hg (oxygenation index = 128). Then he turned to respiratory distress, cyanosis, oliguria, gross edema, and renal deterioration.

In consideration of severe state, ceftazidime combined with moxifloxacin was prescribed, oral prednisone was replaced by intravenous methylprednisolone, and low molecular heparin was injected subcutaneously for anticoagulation. On May 30, though vital signs were stable, urine volume was still 310 mL in day, creatinine zoomed up to 579 μmol/L, far worse than expectation. Continuous renal replacement therapy was started immediately through right femoral vein catheter. Two hundred fifty milliliters of water was removed every hour, and total ultrafiltration volume reached to 3913 mL in 3 days. In the following days, the patient was on the mend with increasing urine, releasing abdominal distension, relieved dyspnea, just ankle edema left. On June 12, he was found to have lowering creatinine (372 μmol/L), with serum albumin of 22.7 g/L, brain natriuretic peptides of 570 pg/mL, procalcitonin of 0.63 ng/mL.

## 3 Discussion

### 3.1 Autosomal dominant polycystic kidney disease

With an estimated incidence between 1:400 and 1:1000 live birth,^[1]^ ADPKD is a congenital disorder primarily affecting kidneys, characterized by the development and growth of cysts, though other organs may be involved, such as liver, gastrointestinal tract, and arterial blood vessels.^[2]^ In most cases, almost 85%, is caused by a mutation localized on chromosome 16 (ADPKD_1_), and nearly 15% by a mutation in chromosome 4 (ADPKD_2_), with a little left unknown.^[3,4]^

Usually in this disorder, urinary protein excretion is less than 1 g/24 hour, while nephrotic syndrome is considered to be rare.^[5]^ The histopathological lesions reported are variously covering focal segmental glomerulosclerosis,^[6]^ membranous nephropathy,^[7]^ minimal change disease,^[8]^ crescentic glomerulonephritis,^[9]^ immunoglobulin A nephropathy,^[10]^ amyloidosis,^[11]^ and mesengioproliferative glomerulonephritis.^[12]^ It proved to be invalid of hormone empirical therapy for the patient with no sign of proteinuria reducing. Though a kidney biopsy is an invasive but meaningful procedure, a new approach combining with computed tomography or laparoscopy is not available.

The most common causes of an acute exacerbation on chronic renal failure in ADPKD patients embrace volume depletion, obstruction, and infection.^[13,14]^ Severe hypertension, gross hematuria, repeated infection, especially in men, and massive kidney size would accelerate progression to end-stage renal disease.^[13,14]^ ADPKD_1_ appears to also have a worse prognosis than ADPKD_2_.^[13]^ This patient was attacked by acute renal failure, acute respiratory distress syndrome, septicemia, which aggravated the state of illness. Acute renal failure due to nephritic syndrome generally is reversible with a favorable prognosis. There may be following mechanisms: Nephrotic-range proteinuria and hypoalbuminemia leads to sodium retention, causing hypovolemia and insufficient renal blood flow; Renal interstitial edema raises the compression of renal tubule, resulting in obstruction; The protein in urine will block the renal tubules.

### 3.2 Hypertrophic cardiomyopathy

HCM, another inherited disorder, is a global disease reporting a prevalence of 0.2% of the general population, or 1 in 500.^[15]^ The generally accepted definition of HCM is a disease state characterized by LV hypertrophy without any obvious cause.^[16]^ Diagnosis is usually by echocardiogram by maximal LV wall thickness 15 mm, particularly in the presence of other compelling information.^[16]^ In fact, fully one-third of patients have no obstruction.^[17]^ The clinical presentation of HCM may also be frequently asymptomatic, until featured by disabling cardiac symptoms, tachyarrhythmia, and electrocardiographic abnormalities.^[18]^ HCM is most commonly due to a mutation in 1 of 8 genes that results in a mutated protein in the sarcoma, for beta-myosin heavy chain, myosin binding protein C, troponin T, troponin I, alpha tropomyosin, actin, regulatory light chain, and essential light chain.^[19]^

There are 3 common complications in clinical progression, sudden cardiac death (SCD), heart failure, and atrial fibrillation.^[18]^ It showed that cardiac arrest or sustained ventricular tachycardia, early onset of family history of sudden cardiac arrest, unexplained syncope, nonsustained tachycardia, exercise-induced low blood pressure under the age of 50, severe LV hypertrophy (>30 mm), cardiac insufficiency (<50%) are main risk factors of SCD.^[16]^ This patient had a history of unexplained syncope without atrial fibrillation, LV wall thickness less than 30 mm, the ejection fraction greater than 50%, but the genetic test was not done. In according to O’Mahony et al the ESC-HC prediction formula for SCD is as follows:

Probability SCD at 5 years = 1–0.998^exp(Prognostic index)^;

where prognostic index = [0.15939858 × maximal LV wall thickness (mm)]

−[0.00294271 × LVmaximal wall thickness^2^ (mm^2^)] + [0.0259082 × left atrial diameter (mm)]

+[0.00446131 × maximal (rest/valsalva) LV outflow tract gradient (mm Hg)] + [0.4583082 × family history SCD]

+[0.82639195 × nonsustained ventricular tachycardia] + [0.71650361 × unexplained syncope]

−[0.01799934 × age at clinical evaluation (years)].^[20]^

On the basis of the score, our patient got score 5.74, implantable cardioverter defibrillator may be considered.^[20]^

Patients with HCM have raised oxygen demand due to the hypertrophy and adverse loading conditions.^[16]^ Aimed to a heart rate of less than 60 to 65 beats per minute, beta-blocking drugs are highly recommended for treatment of symptoms as angina and dyspnea.^[16]^ With the heart rhythm slowing down, myocardial oxygen supply is improved to meet the demand, so that to reduce myocardial ischemia, prolong the diastolic filling period.^[21]^ In consideration of the patient's condition, type I respiratory failure was unable to provide oxygen to the heart muscle, oliguria increased the load on the heart. To be on the safe side, tartaric metolol was increased to maximum tolerable dose, and hemodialysis was used to reduce cardiac capacity.

## 4 Conclusions

In summary, polycystic kidney disease and HCM are both relatively rare diseases. There might have been a chance association, but nephrologists should be aware of this potential association. To our knowledge, this is the first reported case of ADPKD with HCM, with complete remission of acute kidney injury and preservation of cardiac function.
